# Constructing stability - a classic grounded theory of next-of-kin in palliative cancer care

**DOI:** 10.1186/s12904-020-00580-7

**Published:** 2020-06-05

**Authors:** Carina Werkander Harstäde, Anna Sandgren

**Affiliations:** Center for Collaborative Palliative Care, Department of Health and Caring Sciences, Linnaeus University, Växjö, Sweden

**Keywords:** Cancer, Constructing stability, Grounded theory, Next-of-kin, Palliative care

## Abstract

**Background:**

Being next-of-kin to someone with cancer requiring palliative care involves a complex life situation. Changes in roles and relationships might occur and the next-of-kin thereby try to adapt by being involved in the ill person’s experiences and care even though they can feel unprepared for the care they are expected to provide. Therefore, the aim of this study was to develop a classic grounded theory of next-of-kin in palliative cancer care.

**Method:**

Forty-two next-of-kin to persons with cancer in palliative phase or persons who had died from cancer were interviewed. Theoretical sampling was used during data collection. The data was analysed using classic Grounded Theory methodology to conceptualize patterns of human behaviour.

**Results:**

*Constructing stability* emerged as the pattern of behaviour through which next-of-kin deal with their main concern; *struggling with helplessness*. This helplessness includes an involuntary waiting for the inevitable. The waiting causes sadness and frustration, which in turn increases the helplessness. The theory involves; *Shielding, Acknowledging the reality, Going all in, Putting up boundaries, Asking for help,* and *Planning for the inescapable*. These strategies can be used separately or simultaneously and they can also overlap each other. There are several conditions that may impact the theory *Constructing stability*, which strategies are used, and what the outcomes might be. Some conditions that emerged in this theory are time, personal finances, attitudes from extended family and friends and availability of healthcare resources.

**Conclusions:**

The theory shows the complexities of being next-of-kin to someone receiving palliative care, while striving to construct stability. This theory can increase healthcare professionals’ awareness of how next-of-kin struggle with helplessness and thus generates insight into how to support them in this struggle.

## Background

Palliative care is seen as an active holistic care of individuals with serious illness, and especially of those near end of life. The aim of palliative care is to improve quality of life of patients, their families and their caregivers [1].

Being the next-of-kin to someone with cancer who requires palliative care entails a complex life situation. The next-of-kin can experience changes in roles and relationships and try to adapt by actively being involved in that person’s experience and, even if it is difficult, try to maintain a positive outlook [2]. Living with incurable cancer implies that life is drawing towards its end, which can cause great conflicts both for patients and next-of-kin as it resembles a finitude that is difficult to adapt to [3]. However, next-of-kin can also have positive experiences when caring for the person such as enhanced self-esteem and that they are playing an essential role in the person’s care [4]. Still, next-of-kin can feel unprepared for the care they are expected to provide as they may not have the knowledge and/or competence to provide this care [5]. They can experience a burden as they adjust their own schedules and relinquish personal activities and time [6] thus putting their own lives on hold [7]. Living with a person with cancer affects the whole family and can also increase the next-of-kin’s risk of becoming ill themselves [8].

Still many next-of-kin choose to be involved in caring for the ill person and the reasons for doing so can vary. Some may have a personal desire to give back some of the care and support that they themselves have received; however, the reason may also include maintaining family autonomy or a wish to avoid expected low quality of care or uncoordinated formal service [9]. The ill person can also directly or indirectly rely on the next-of-kin to provide consistent care and most often, they appreciate this involvement [10]. Nonetheless, the next-of-kin can feel exposed in these situations and perceive that they are not ‘up to the task’, either in their own eyes or in the eyes of others [5]. This might evolve into feelings of guilt and/or shame [11, 12]. Coming face-to-face with the forthcoming death can also result in bitterness towards losing a lot of what is important in life [13]. However, it can also be a reminder of what is essential, which may lead to reprioritising and enjoying every moment with the ill person and even appreciating him/her more [14]. It can also feel like of a meaningful endeavour despite its challenges [15]. Next-of-kin have to adjust to different situations where they often have the main responsibility for the person’s care. This place them in a unique situation, where they both give support and care, at the same time as they might be in need of support and care [16].

Previous research describes how next-of-kin experience their situation in palliative cancer care; it is therefore of importance to elaborate the understanding of how the next-of-kin handle this complex situation. Therefore, the aim of this study was to develop a classic grounded theory of next-of-kin in palliative cancer care.

## Methods

The methodology used in this study was classic grounded theory, developed by Glaser and Strauss [17] and further outlined by Glaser [17–21]. Grounded theory was chosen since it aims to conceptualise patterns of human behaviour.

This study was conducted between 2017 and 2019. Data from 42 interviews with next-of kin were coded and analysed. At first, nine next-of-kin to persons with cancer in late palliative phase or persons who had died from cancer were interviewed. Six were women and three were men; age 35–82 years (mean age 58). Their relationship to the ill persons were husbands, wives, cohabitants, sons, and daughters. The next-of-kin were asked to participate in this study by a palliative home care-team in southern Sweden that was or had been involved in the care of the ill person. If they were interested they gave their permission for the researcher to contact them. The inclusion criteria were that they were closely related to and took/had taken care of a person with cancer in palliative stage. Ten next-of-kin were asked to participate and nine agreed. The reason for not wanting to participate is not known, neither is this person’s relation to the patient known.

All interviews were performed in participants’ homes except one, which was performed in a coffee shop upon the next-of-kin’s request. The interviews were informal, open-ended conversations and the next-of-kin were encouraged to speak openly about their situation with the person who was dying from cancer or had recently died from cancer. Open ended questions were asked; for example, ‘Can you tell me about your situation as a next-of-kin’ and’ Can you describe your feelings when this happened’. The interviews were thus more like open conversations than formal interviews. The interviewer (CWH), who is well experienced in performing qualitative interviews, was sensitive and open towards what the next-of-kin narrated.

The interviews lasted 60–120 min. Field notes were written during and after each interview and served as the basis for data analysis. Alongside the data collection and analysis, memos were written to keep track of ideas about main concern and the connections amidst emerging concepts [18, 20].

The field notes were used in the generation of concepts, which commenced with an open coding of the data. This involved analysing the text line-by-line as soon as possible after each interview, by asking different questions: What do these data reflect? What category does this incident indicate? What is truly happening in these data? What is the next-of-kin’s main concern? and How do they continually handle this concern? By asking these questions, the researcher elucidated the properties that repeated themselves in the data instead of focusing on solitary events.

Alongside this analysing, memos were written to gather thoughts and ideas about the codes and their relationships and to draw out theoretical properties of the codes. This was done to reach saturation and help define boundaries of the codes [18]. The open coding process was accompanied by constant comparison where initial codes were compared with each other as they emerged, newly generated concepts were compared to new codes, and concepts were compared to other concepst. Theoretical sampling [18, 20] was used to guide the interviews so that new ideas that emerged during interviews and the analysis could steer the following interviews to saturate those ideas. Instead of considering sample representativeness, grounded theory aims to collect data to refine and elaborate emerging concepts and focus on concepts related to the core concept and emerging theory [18, 20]. For example, a strive was to recruit next-of-kin who were related in different ways to the ill person so that emerging ideas could be more and more saturated. New and more specific questions to ask emerged during the analysis process, for example: “How are you able to handle the situation?” and “How does it feel to ask for help” These questions were then used in subsequent interviews, to saturate the concepts in the theory. Saturation was reached when the coding did not contribute with any further concepts or properties in relation to the core concept.

When the core concept had emerged in the open coding phase, it was followed by the selective coding phase in which collected data and codes were defined into accurate concepts related to the core concept. A secondary analysis was done on 33 transcribed interviews, previously conducted with next-of-kin to patients who had died from cancer [11, 12]. These persons were over 18 years old and lived together with the ill persons, their relation were husbands (*n* = 3), wives (*n* = 17), cohabitees (n = 3), son (n = 1) and daughters (*n* = 9). The purpose of the secondary analysis was to compare and saturate the emerging concepts. Only the concepts that were related to the core concept were included in the analysis. Throughout the continuing analysis, more memos were written about the concepts and their relationships to each other and the core concepts. Memo sorting was performed to continue to track ideas [20]. Coding continued until theoretical saturation was accomplished; this means that no new concepts emerged in the analysis of new data.

In the next phase, theoretical coding, relationships between the concepts and the core concept emerged through sorting of the written memos. Further, memos on memos were written to maintain on conceptual (rather than descriptive) level. The theory was then written up, and the elements of time, place and people were left behind, according to Glaser [20]. It should be noted that the theory is not the voice of the participants; rather it is a generalisation of the actions of individuals with conceptual hypotheses of the behaviour emerged in the research area.

### Ethical considerations

Ethical approval for the study was obtained from the Regional.

Ethical Board in Linköping, Sweden (2014/304–31). All participants received written and oral information about the aim of the study and the possibility of withdrawing their participation at any time without need to give reasons for doing so. Verbal and written consent was obtained from each participant as a way to make sure that they understood what the participation in the study meant, and this complied the ethical board’s request. Confidentiality was assured according to ethical research guidelines. Informed consent was obtained from all participants [22]. Personnel from the palliative home care team asked the proposed participants if they would consider taking part in the study and informed them that it was voluntary. If they agreed, contact information was given to the researcher who then contacted them. In that way the proposed participants did not have to speak directly to the researcher and state their decision if they did not want to participate, something that might have caused them difficulties.

## Results

Next-of-kin in palliative care are consistently living with the knowledge of an imminent and inevitable loss of the ill person. Since they cannot influence the outcome of this situation, they often feel helpless and powerless. *Struggling with helplessness* emerged in this grounded theory as the main concern for next-of-kin. This helplessness includes an involuntary waiting for the inevitable. The waiting causes sadness and frustration, which in turn increases the helplessness. Feeling helpless is brutal and extremely stressful and has an impact on all involved. Since it is difficult and energy-draining to consistently live in helplessness, the next-of-kin need to evade this situation to emotionally survive and return to normalcy, the everyday things that are always going on. This is accomplished by handling the situation with different strategies; although it might not make a difference in the long run, the ill person will still die.

The theory *Constructing stability* explains the pattern of behaviour through which next-of-kin handle their complex struggle with helplessness. In *Constructing stability*, there is a wish to create an everyday normal life that is solid ground, which helps the next-of-kin go on. The theory involves the following strategies: *Shielding*, *Acknowledging the reality*, *Going all in*, *Putting up boundaries*, *Asking for help*, and *Planning for the inescapable*. There are several conditions that can influence the theory and there are also several feasible combinations of the strategies in the theory. The strategies can be used separately or simultaneously; as they are not always completely detached, they can be mixed and overlap each other, depending on the often complex circumstances. Depending on the different combinations of strategies, there are different theory outcomes. Some strategies may help next-of-kin to move on while some may leave them feeling even more frustrated and struggling.

### Shielding

When using *Shielding*, imaginary blinders are used consciously or unconsciously, which hide what is ‘actually’ going on. Next-of-kin hold on to the notion that ‘everything is fine’, nothing is wrong and that life is going on exactly as it always has. In *Shielding* the next-of-kin are in a vulnerable state. *Shielding* is like a soap bubble that easily bursts by trigger points like a sudden impairment of the ill person, a visit to the hospital, or when the next-of-kin starts thinking about what lies ahead and realise that they have been living in denial. Seeing the progress of the illness does not meld with the *Shielding* strategy. The next-of-kin come to a dividing line and must ask themselves what is most important; to live in denial or to face reality. Persistently blocking out reality will eventually lead to a situation in which the ill person is also shut out. *Shielding* can thus be difficult to maintain over time. However, if the next-of-kin can maintain this strategy, the consequence is disconnection from veracity, which leads to no need for other strategies. By facing the truth, next-of-kin may shift to the strategy *Acknowledging the reality*.

### Acknowledging the reality

The strategy *Acknowledging the reality* can be used after *Shielding* or it can be used directly when realising what is going on with the ill person. It means accepting the situation and not turning away from the reality that the ill person is not going to get well again. When the next-of-kin do so, they try to find strength in doing the familiar and ordinary things together with the ill person; in other words, maintaining a life that is not changed by the illness, or at least, affected as little as possible. It is a way of trying to embrace life to the fullest. This strategy follows the next-of-kin through the course of events unless they shift back to *Shielding* or shift into *Shielding* for the first time since the reality is too much to take.

### Going all in

In *Going all in*, the next-of-kin are doing as much as possible to support the ill person. There is nothing that can stop them and they sometimes end up being with the person 24/7, with no time at all for themselves. *Going all in* can thus be immensely demanding, leaving no time for the next-of-kin to reflect on their emotions. Time for themselves is not considered important. It is a choice they make, to be there merely for the ill person. As a husband, wife, or child you are expected to *go all in*. This decision can be based on a pure act of love or it can be based on a reasoning that it is the only moral thing to do. *Going all in* can lead to next-of-kin falling ill themselves. The next-of-kin can be so tired but see no alternative but to be there by the ill person’s side, no time to feel, just being in the situation. This is often overlooked at first, and sometimes not taken seriously until the person has died. Worries about how time will last and about one’s personal finances can also affect how the next-of-kin handle the situation.

*Going all in* involves *Hanging in there*, which means following the ill person in the ‘ups and downs’ and constantly being by his/her side. This can create wonderful moments with possibilities to capture the small things in life, like seeing the birds outside in the birdhouse, taking a short walk, or preparing dinner. Capturing moments to remember is important to minimise helplessness. However, *Hanging in there* can also involve moments of despair, as it connects to how the illness is progressing; for example going to a doctor’s appointment and receiving sad information.

*Going all in* can further involve *Standing up for*, since the next-of-kin feel the need to step up for the ill person. This is owing to the perception that the person is not receiving acceptable care. It can be about the home care service not working properly for example not getting food, lack of medication, not being helped to the toilet and so on. The next-of-kin then try to compensate for the absence of care and be with the ill person all the time. They also pressure involved health professionals to ensure that everything is ‘done right’ for the person. *Standing up for* is also used when the ill person does not have the strength to speak up for himself/herself. The next-of-kin then acts as a spokesperson.

### Putting up boundaries

Contrasting *Going all in*, *Putting up boundaries* is used when putting time into being together with the ill person is considered more important than basic caregiving needs or household work. *Putting up boundaries* can also be a way to not use the strategy *Going all in*, if the demands are too much. It is a reasoning with oneself that emerges from an insight into what is most important in the complex situation: to persist through the course of events i.e. the person’s illness and dying, *Putting up boundaries* and persisting through the opinions of others requires strong belief that the next-of-kin maintain their points of view concerning what is most important. If that is not possible, they can instead end up using the strategy *Going all in* once again or for the first time.

*Putting up boundaries* involves *Keeping roles intact* and *Saving one’s strength*. When *Keeping roles intact*, the next-of-kin are very clear and firm about what they will and will not do when it comes to being involved in the ill person’s care. Choosing not to help the person with personal hygiene, changing diapers, showering etc. can be a way of keeping the family roles intact; for example, by saying: ‘I am a wife and not a care worker’. *Saving one’s strength* by requesting help with cleaning, washing, and cooking to enable quality time with the ill person is also a *Putting up boundaries* approach.

### Asking for help

If next-of-kin are on the continuum from *Going all in* to *Putting up boundaries*, the strategy *Asking for help* will be increasingly visible. It evolves from the fact that the next-of-kin often strive to handle the entire situation by themselves as long as possible, by *Going all in*; however, sometimes this is not conceivable. They wait until there is no return since it is often difficult to show helplessness. Next-of-kin can thus be reluctant to ask for help; however, they eventually realise that they must. They cannot handle the situation without help. The closer they get to *Putting up boundaries* the more this asking might turn into a demand; however, before that, the next-of-kin struggle with showing that they are not able to handle the situation on their own.

*Asking for help* from the extended family or outside the family can feel like a failure; however, it can also be a relief because next-of-kin experience compassion and a willingness of others to ease their many times complex burdens. Moreover, if the next-of-kin have positive relationships with healthcare providers, other relatives, and friends and if they feel that they can discuss their situation without being questioned, *Asking for help* might not be needed. The help comes because it is noticed what is needed and by doing that, the help is arranged. To get help can thus be a comforting surprise when next-of-kin realise that they are not alone.

*Asking for help* can however also lead to being questioned by healthcare providers, other relatives, and friends, if they really are doing a good enough job when caring for the ill person. This in turn, leads to feelings of despair and feelings of ‘not being good enough’, which are hard to reconcile. Next-of-kin might then return to *Going all in* again.

### Planning for the inescapable

Through the whole situation of being next-of-kin in palliative care there is also a need for *Planning for the inescapable*, which means facing the ill person’s forthcoming death; for example to discuss how funeral arrangements should be arranged, how personal financial matters, and so on should be sorted out. To have conversations with the person about what will happen during the nearest future, but also what will happen after the death. Although what lies ahead is cruel and sad, talking about it can be a relief. *Planning for the inescapable* is firmly connected with the ill person’s inevitable death. This is a strategy that some families use early on during the course of illness. However, not all next-of-kin, nor the ill persons, can handle these conversations. They can be too overwhelming and might cause too much sorrow to the persons involved; therefore, this strategy might be avoided. The next-of-kin might instead employ *Shielding*.

Time aspects in *Planning for the inescapable* can be hard to determine; however, it is explicit that the closer to death the person gets the more inevitable this talking and planning becomes. It is a way of *Acknowledging the reality* and awaiting the death to come. It is a paradox; in one way, the death is not wanted; however, in another way, it is something to look forward to, as an end of suffering. Although this is dichotomously, the next-of-kin can see the forthcoming death in both these ways simultaneously. They are struggling with wanting to keep the ill person alive and letting him/her go concurrently. This might lead to a guilty conscience or sorrow as the next-of-kin often find themselves alone with their struggle since they feel that they cannot share their thoughts with the ill person and cannot *Plan for the inescapable* together. If they feel that they can share these thoughts with the person this planning might instead be a way to reach mutual understanding where they can support each other.

### Conditions influencing the theory constructing stability

There are several conditions that may impact the theory *Constructing stability*, which strategies are used, and what the outcomes might be. Some conditions that emerged in this theory are time, personal finances, attitudes from extended family and friends and availability of healthcare resources.

Having time is something that next-of-kin, if possible, try to plan by choosing what to do themselves and what to ask for or demand help with. Time is also connected to financial matters, as being able to spend time with the ill person without having to think about financial aspects may reduce helplessness. Thus, not having money or worrying about having enough money by having to be on leave from work may negatively affect the next-of-kin’s caregiving. These conditions are easier to handle for the next-of-kin who are retired since their finances are less affected compared to those who have to work. Further next-of-kin who run their own businesses can adjust their working time; however, they often lose opportunities or will eventually need to catch up on work that is missed.

Extended family and friends can be a most welcome support if the next-of-kin feel comfortable with their involvement; however, there is a difference between being helped and being questioned concerning the care of the ill person. If the next-of-kin feel judged by extended family and/or friends it can lead to feelings of inadequacy or shame.

Some next-of-kin, who themselves work in healthcare or have previous healthcare experiences feel that they have a shorter way into healthcare services. Knowing what to do and knowing who to turn to when help is needed facilitates the situation. However, next-of-kin also feel taken advantage of when healthcare providers know about their experiences: they do not receive information, or they are assumed to be able to handle things on their own without being properly asked if that is okay. Having healthcare knowledge is also a hindrance because the next-of-kin might feel that the care the ill person is receiving is insufficient.

Next-of-kin strive to do the best they can in *Constructing stability*, regardless of the time and cost restraints and they may find themselves at a continuum between two extreme poles: *Going all in* and *Putting up boundaries*. Some end up *Going all in*, which might involve *Going with the flow* and/or *Standing up for*, while others are *Putting up boundaries* that might involve *Keeping roles intact* and/or *Saving one’s strength*; however, most move back and forth between these poles.

### Feasible combinations of theory strategies

The different strategies in the theory can, as mentioned before, be used separately or simultaneously; however, some combinations are impossible (see Table 1).

**Table 1:** Feasible combinations of constructing stability strategies

	Shielding	Acknowledging the reality	Going all in	Hanging in there	Standing up for	Putting up boundaries	Keeping roles intact	Saving ones strength	Asking fo help	Planning fot the inescapable
Shielding		–	–	–	–	+	–	+	–	–
Acknowledging the reality	–		+	+	+	–	+−	+−	+	+
Going all in	–	+		+	+	–	–	–	–	+
Hanging in there	–	+	+		+	–	–	–	+	+
Standing up for	–	+	+	+		+−	+−	+−	+−	+
Putting up boundaries	+	+−	–	–	+−		+	+	+	+
Keeping roles intact	–	+−	–	–	+−	+		+	+	+
Saving ones strength	+	+−	–	–	+−	+	+		+	+
Asking fo help	–	+	–	+	+−	+	+	+		+−
Planning fot the inescapable	–	+	+	+	+	+	+	+	+−	

+ = strategies can be combined, − = strategies cannot be combined, +/− = strategies can or cannot be combined depending on circumstances

For example, next-of-kin can never use the strategy *Shielding* while simultaneously adopting any of the strategies that include acknowledging the reality. However, strategies that are concerned with *Putting up boundaries*, and avoidance can be combined with *Shielding*.

There are also combinations that can both work and not work depending on the circumstances. For example *Standing up for* can be combined with *Keeping roles intact* and *Saving one’s strength*. When next-of-kin are *Standing up for* this may be to keep roles intact so that healthcare providers do not make all the decisions in the care of the ill person. *Standing up for* can, on the other hand be impossible to combine with *Keeping roles intact* since it involves a shift in the family, where the ill person no longer can speak for himself/herself. *Standing up for* can also be utilised to save strength. To have a say in what is needed in care and how this care should be given can lead to a mutual understanding between next-of-kin and healthcare providers, which lead to cooperative relationships that is strength saving. *Saving one’s strength* can also be difficult to combine with situations where the next- of kin stands up for the ill person. *Standing up for* can demand a lot of effort, making it impossible to *save one’s strength*.

### Feasible outcomes of the theory

There are several outcomes of the theory; however, two outcomes appear explicitly. To help and support the ill person can sometime ease the helplessness that is felt. To do something worthwhile and to be there can create *Feelings of being honoured* and make a difference in the midst of helplessness. It provides strength that is needed through the course of the illness and after the person has died. However, it is not always possible for the next-of-kin to have the strength to be this helpful which leads to *Feelings of frustration* when inability to handle the situation occurs. Next-of-kin sometime handle this frustration by expressing anger, which can be aimed at the ill person, healthcare providers, other family members, or internally. It can also have no aim and rather just cause them to handle the situation hastily. The anger does not lead to any constructive solutions; instead, it often leads to the next-of-kin having a guilty conscience, attempting to compensate by *Going all in*.

## Methodological considerations

This study used a classic grounded theory to explore the main concern of next-of-kin in palliative care and how they handle this concern. The theory that emerged – *Constructing stability* - explains the latent behaviour and elucidates of the strategies that next-of-kin utilize to cope with feelings of helplessness. However, the theory cannot claim to be representative of next-of-kin’s entire behaviour during palliative care in all contexts. Nonetheless it should be emphasized that grounded theory generates hypotheses that are conceptual, and abstract of time, place, and people [20]. This means that although this theory is in the field of palliative care, it can be applied and modified to other areas, for example, when people feel helpless during radical life changes.

The first nine interviews were not recorded and transcribed verbatim, which could be discussed because field notes do not cover all details in the interviews. However, in classic grounded theory, the focus is on common concerns and repeated patterns in the data, and recorded and transcribed interviews may increase the risk of focusing more on individual details than on repeating patterns of behavior in the data [18, 20].

According to Glaser [18], a classic grounded theory should be judged by its fit, relevance, workability, and modifiability. Fit means how well the concepts fit the data that they are representing. To achieve this, no pre-existing theoretical perspective or preconceived concepts were used; instead, all the concepts emerged from the collected data through constant comparison. Relevance means that the emerged concepts are related and relevant to the main concern. To address relevance, the data were collected and analysed until saturation was obtained. Both authors were involved in the analysis, however the first authors did the main coding of the data. The concepts and the emerging theory was also discussed in a palliative care research group. The theory has been shown to be applicable to the studied area when presenting the theory at workshops. Workability means that the theory explains how the main concern is resolved. Therefore, the analysis was focused on finding a core concept that explains how the main concern is resolved with as much variation as possible. Lastly, the criterion modifiability concerns how the theory can be modified with new data as mentioned above.

## Discussion

*Struggling with helplessness* emerged as the main concern. Similar concerns have been described in former studies where experiences of next-of-kin in palliative care are presented, although, this knowledge is limited [23]. Foran Lewis [24] showed how next-of-kin in dementia care try to handle being trapped in an inescapable role by sacrificing themselves and trying to reclaim identity. These strategies of taking care of an ill person are in many ways similar to *Going all in* or *Putting up boundaries* in our theory. There are also similarities when it comes to the strategy *Acknowledging the reality*, where the attempts to live a ‘normal everyday life’ can be compared to ‘Living in the moment’ [24] and striving for normality [25]. The next-of-kin know what lies ahead for the ill person; however, at the same time they cannot foresee when this will happen. This uncertainty leads them to either ignore or face reality, which becomes more prominent when patients themselves talk about their forthcoming death [26]. Healthcare professionals can help next-of-kin in their efforts to have an everyday life by facilitating contacts between hospital and home and being available to support when there is a risk that next-of-kin’s energy might run out. Just knowing that help is accessible can sometimes in itself be enough for next-of-kin to carry on even if the situation is strenuous.

For both patients and next-of-kin there seems to be a need to, at least for a while, shut out what is happening. This way of handling the situation is seldom long-lasting and seems more like a way of masking the reality than adapting to what is going on. Concurrently, it is important to be able to catch one’s breath since the experience can be overwhelming and demand so much energy. Therefore, *Shielding* can be a respite. Consistently, Bruce, Schreiber, Petrovskaya and Boston [27] describe it as a process of longing for ground in a groundless world, which includes turning away from the distress by keeping it out of consciousness. It can also be compared to an oscillating process where the next-of-kin find themselves between the need to be close to the ill person and the need for distance from the pain of the coming loss [28].

Acknowledging the reality can be compared to Sandgren et al. [7] findings about how patients receiving palliative care and their next-of-kin struggled for a total presence in the here and now by trying to optimise life by taking every chance to be happy. Sometimes next-of-kin might need help and encouragement from healthcare professionals to realise that it is okay to seek positive energy. That feeling, good for a while, should not be followed by feelings of guilt for not taking the illness seriously. Healthcare professionals should also seek ways to facilitate in the care of the loved one so that next-of-kin can put time and energy into optimizing life here and now.

Next-of-kin’s situation is often overlooked since all attention is aimed towards the patient. This is important to recognise as next-of-kin described feelings of not being listened to and having to struggle to get the care that was needed. In a review of research involving next-of-kin who provided care for patients with chronic illnesses it was found that they were at risk of caregiver burden, which could result in depression, social isolation, and financial stress [29]. Stress and depressive symptoms were also found when examining caregiver burden among next-of-kin in palliative care [8], as well as next-of-kin to patients with late-life depression [30]. Being next-of-kin in palliative care is complex and demanding; it comprises changing roles and novel practical and emotional challenges [31]. Next-of-kin can also be uncertain regarding their own needs, as they put the patient first [32]. Andershed [33] describe a balance between burden and capacity. The will to do what is best for the ill person might place the next-of-kin in a position where they become ill themselves since they disregard their own wellbeing. It is thus vital for healthcare professionals to be attentive towards how next-of-kin are feeling in their vulnerable situation. They often want to take care of the ill person as much as possible; in doing so they often forfeit taking care of themselves. Psychosocial interventions such as support groups and psychoeducational interventions can be a way to recognise next-of-kin’s needs [29].

The theory shows that preparing for the future is not easy; however, it can be facilitated if both the next-of-kin and the ill person are able and willing to talk about what lies ahead. The person’s incurable illness takes the next-of-kin into a world where there is a need to live in the present and try to savour each moment. Concurrently, the next-of-kin can find themselves focusing on the present and preparing for the future [34]. It is not always easy to start conversations about this unwanted situation; however, healthcare professionals can play a key role in facilitating such conversations by being perceptive towards what the next-of-kin need. Research has also shown the need for healthcare professionals to direct attention towards next-of-kin and their specific needs for support; for example, how they should prepare for the ill person’s end-of-life period, practical arrangements and regular contact [35].

## Conclusion

The theory *Constructing stability* involves several strategies that explain next-of-kin’s behavioural patterns when *struggling with helplessness*. It shows the complexities of being the next-of-kin in palliative care.

The theory can increase the healthcare professionals’ awareness of how next-of-kin cope with their struggle with helplessness and can generate insight into how to help them in their struggle. To be seen and heard is essential; therefore, healthcare professionals should assess and thereafter tailor the support per next-of-kin. The theory can thus contribute to healthcare professionals being better prepared to meet the next-of-kin’s needs and foresee how they should act. *Constructing stability* can also contribute to a general understanding of complex life situations and how people cope with helplessness in other contexts. However, further research needs to elaborate how healthcare professionals can provide optimal care to meet next-of-kin’s needs. Further research is also needed concerning how *Constructing stability* can be facilitated for next-of-kin in palliative care by interconnecting the attitudes and viewpoints of patients, next-of-kin, family, friends, and healthcare professionals.

## Data Availability

The dataset generated and analysed during the current study is not publicly available to maintain confidentiality, but it is available from the corresponding author upon reasonable request.

## Abbreviation

GT: Grounded Theory
